# Supplementary material for: K18-hACE2 mice develop respiratory disease resembling severe COVID-19
Source: bioRxiv. 2020 Aug 11:2020.08.11.246314. Preprint. [Version 1] doi: 10.1101/2020.08.11.246314 (PMC7427137; doi:10.1101/2020.08.11.246314)
Supplement: 1 [file NIHPP2020.08.11.246314-supplement-1.pdf]

bioRxiv preprint doi: https://doi.org/10.1101/2020.08.11.246314. this version posted August 11, 2020. The copyright holder for this preprint (which was not certified by peer review) is the author/funder. This article is a US Government work. It is not subject to copyright under 17 USC 105 and is also made available for use under a CC0 license.

Supplementary Figures

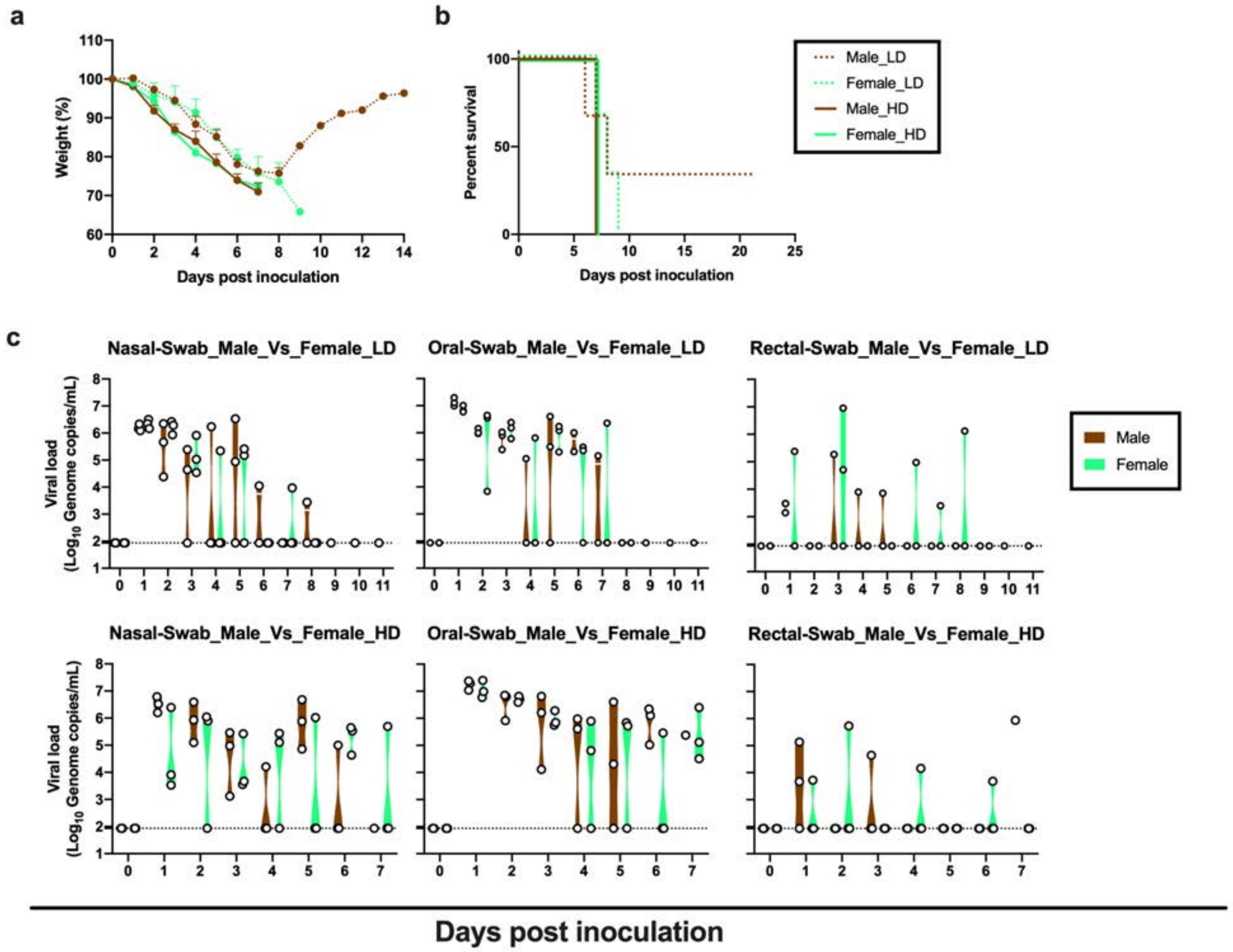

S1 Fig. Sex-dependent weight loss, mortality and virus shedding in K18-hACE2 mice after SARS-CoV-2 infection

bioRxiv preprint doi: https://doi.org/10.1101/2020.08.11.246314. this version posted August 11, 2020. The copyright holder for this preprint (which was not certified by peer review) is the author/funder. This article is a US Government work. It is not subject to copyright under 17 USC 105 and is also made available for use under a CC0 license.

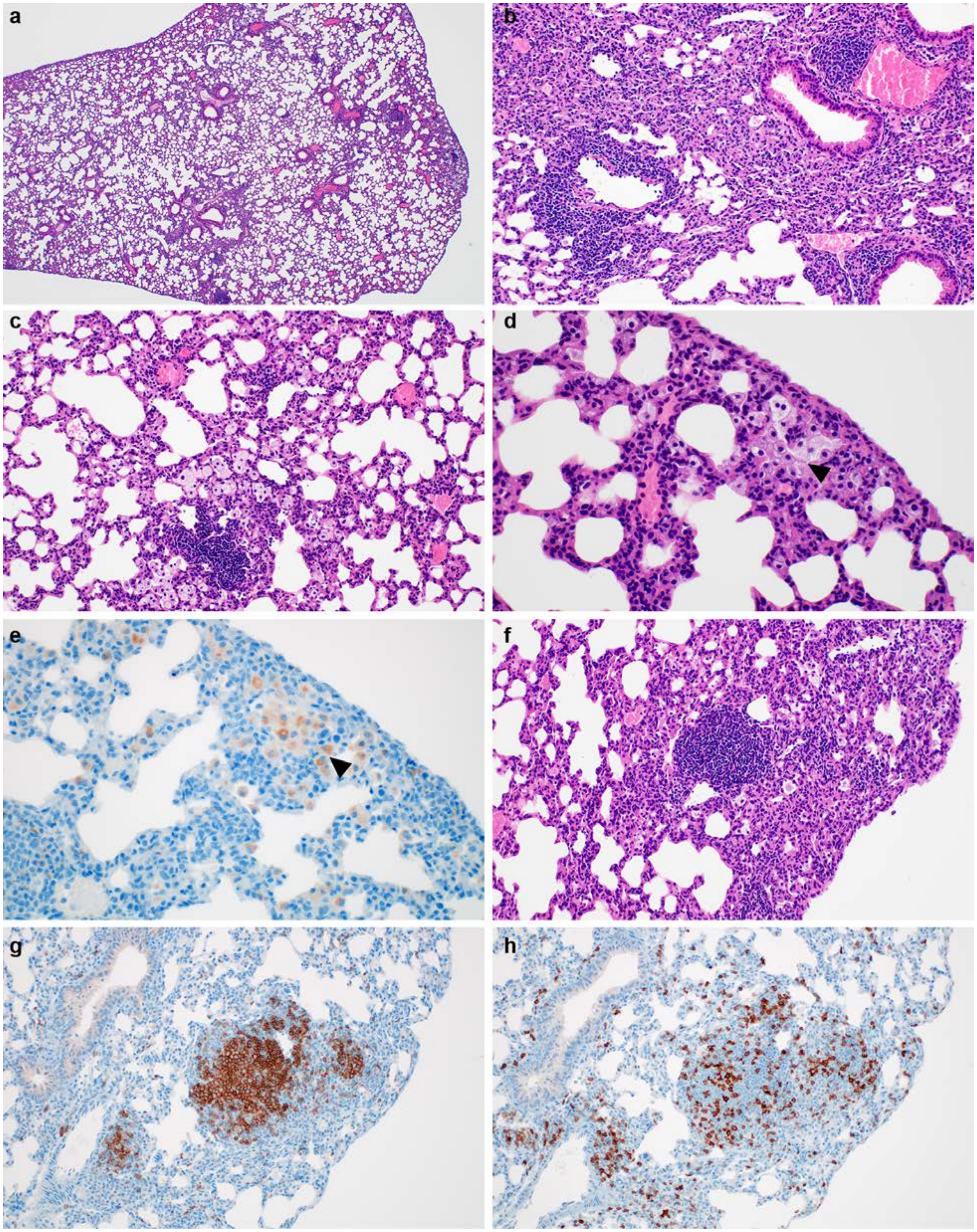

S2 Fig. Histological analysis of lung sections from one low dose survivor at 21 days post infection

bioRxiv preprint doi: https://doi.org/10.1101/2020.08.11.246314. this version posted August 11, 2020. The copyright holder for this preprint (which was not certified by peer review) is the author/funder. This article is a US Government work. It is not subject to copyright under 17 USC 105 and is also made available for use under a CC0 license.

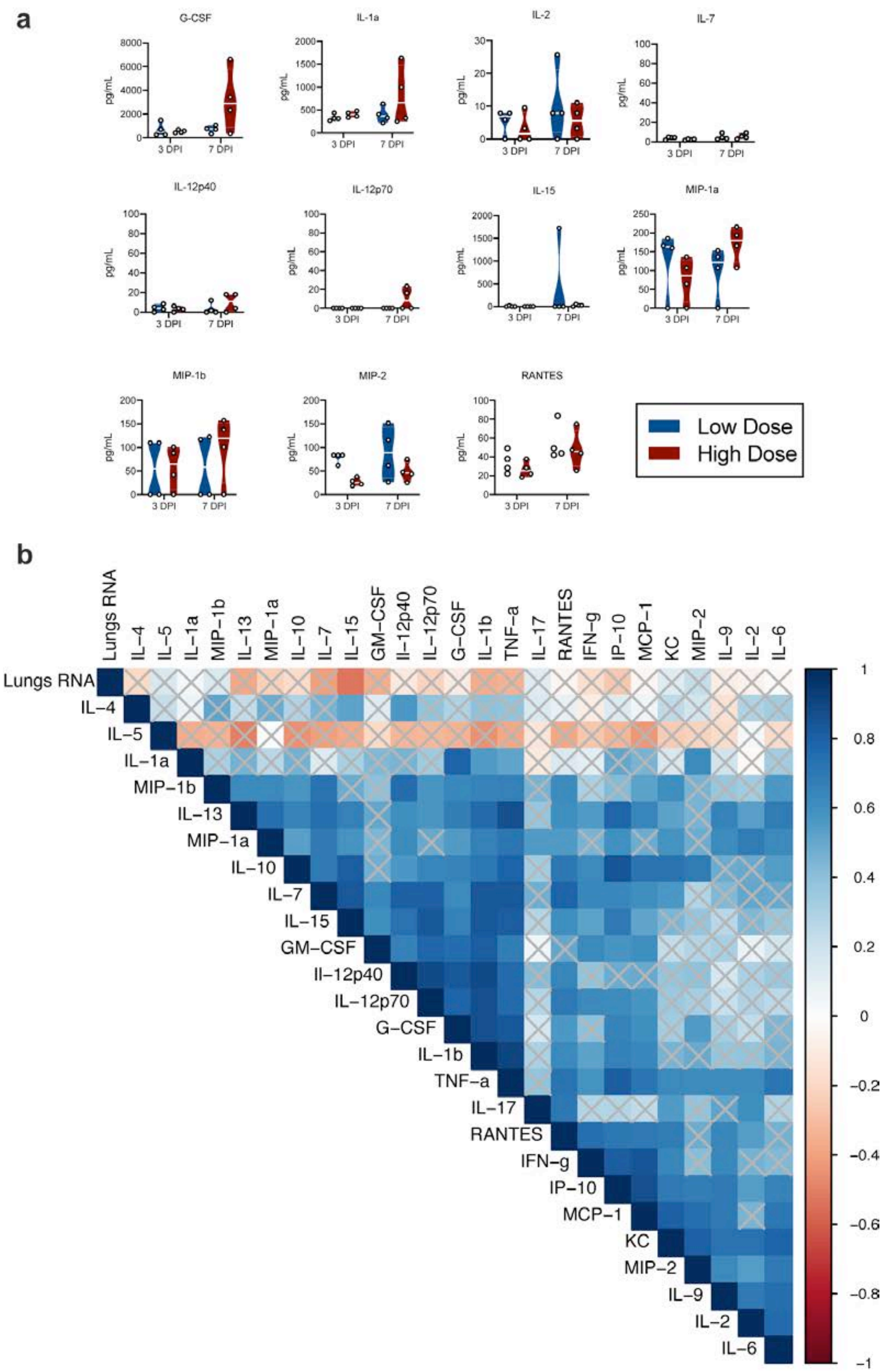

S3 Fig. Multiplex analysis of cytokines/chemokines in K18-hACE mice challenged with SARS-CoV-2

bioRxiv preprint doi: https://doi.org/10.1101/2020.08.11.246314. this version posted August 11, 2020. The copyright holder for this preprint (which was not certified by peer review) is the author/funder. This article is a US Government work. It is not subject to copyright under 17 USC 105 and is also made available for use under a CC0 license.

Supplementary Figure legends

S1 Fig. Sex-dependent weight loss, mortality and virus shedding in K18-hACE2 mice after SARS-CoV-2 infection. a. Body weights were monitored every day. Relative body weight changes are show for female (turquoise) and male (brown) animals for HD (solid) and LD (dotted) groups. **b.** Survival is show for female (turquoise) and male (brown) animals for HD (solid) and LD (dotted) groups. **c**. Nasal, oral and rectal virus shedding in low and high dose infected female (turquoise) and male (brown) mice was quantified by RT-qPCR across time. Individual animals are plotted, violin plot depict median and quantiles. Abbreviations: LD = low dose (10^4 $TCID_{50}$ SARS-CoV-2), HD = high dose (10^5 $TCID_{50}$ SARS-CoV-2).

S2 Fig. **Histological analysis of lung sections from one low dose survivor at 21 days post infection**. **a**. Multiple foci of perivascular inflammation and increased alveolar cellularity. **b**. Perivascular and peribronchiolar lymphocytic inflammation. **c**. Aggregated lymphocytes within alveolar septa and alveoli containing foamy macrophages. **d**. Foamy macrophages cluster and fill alveoli (arrowheads) and alveolar septa contain increased numbers of lymphocytes. **e**. CD68 immunoreactivity in foamy alveolar macrophages (arrowheads). **f**. One of many discreet aggregates of lymphocytes in the 21 DPI lung composed of **g**. CD45+ B cells and **h**. CD3+ T cells. Magnification: a = 40 x; b, c, f, g, h = 200 x; d, e = 400 x.

S3 Fig. Multiplex analysis of cytokines/chemokines in K18-hACE mice challenged with SARS-CoV-2 measured at 3- and 7-days post inoculation. **a**. Individual animals are plotted, violin plots depict median and quantiles. Low dose = blue, high dose = red. **b**. Correlation between cytokine levels and viral RNA in the lungs. Significant correlations (p = 0.05) are shown and strength of correlation is depicted according to the colour bar, crossed bars are not significant. Abbreviations: DPI = days post inoculation, G-CSF = granulocyte colony-stimulating factor, GM-CSF = granulocyte-macrophage colony-stimulating factor, INF = interferon, IL = interleukin, KC = keratinocyte chemoattractant, MCP = monocyte chemoattractant protein, MIP = macrophage inflammatory protein, IP = interferon-γ-inducible protein, TNF = tumour necrosis factor.
